# Supplementary material for: Recurrent breakpoints in the BRD4 locus reduce toxicity associated with gene amplification
Source: Cell Genom. 2025 Mar 19;5(4):100815. doi: 10.1016/j.xgen.2025.100815 (PMC12008804; doi:10.1016/j.xgen.2025.100815)
Supplement: Document S1. Figures S1–S5 [file mmc1.pdf]

Cell Genomics, Volume *5*

Supplemental information

Recurrent breakpoints in the *BRD4* locus reduce toxicity associated with gene amplification

Jeremiah Wala, Simona Dalin, Sophie Webster, Ofer Shapira, John Busanovich, Shahab Sarmashghi, Rameen Beroukhim, Pratiti Bandopadhayay, and Veronica Rendo

Supplemental Information

Recurrent breakpoints in the *BRD4* locus reduce toxicity associated with gene amplification

Jeremiah Wala, Simona Dalin, Sophie Webster, Ofer Shapira, John Busanovich, Shahab Sarmashghi, Rameen Beroukhim, Pratiti Bandopadhayay, Veronica Rendo

Figures S1–S5

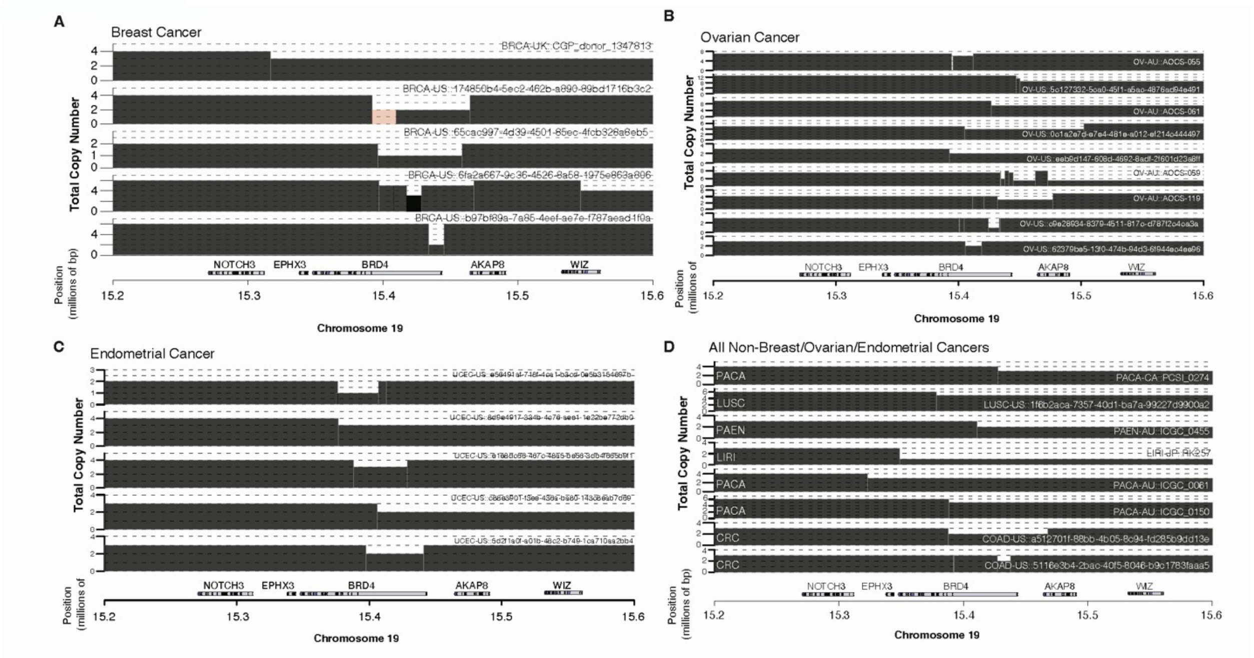

Figure S1. SCNA events at the *BRD4* locus corresponding to the recurrent breakpoints, related to Figure 1. Total copy-number for tumors with either a focal deletion at the *BRD4* locus or an SCNA breakpoint within the *BRD4* locus for **(A)** breast cancer (n=5) **(B)** ovarian cancer (n=9) **(C)** endometrial cancer (n=5) and **(D)** all other cancers including pancreatic adenocarcinoma (PACA; n=3), pancreatic neuroendocrine tumor (PAEN; n=1), lung squamous cell carcinoma (LUSC; n=1), hepatocellular carcinoma (LIRI; n=1) and colorectal cancer (CRC; n=2). Only those tumors with *BRD4* focal deletions are included in subsequent analyses.

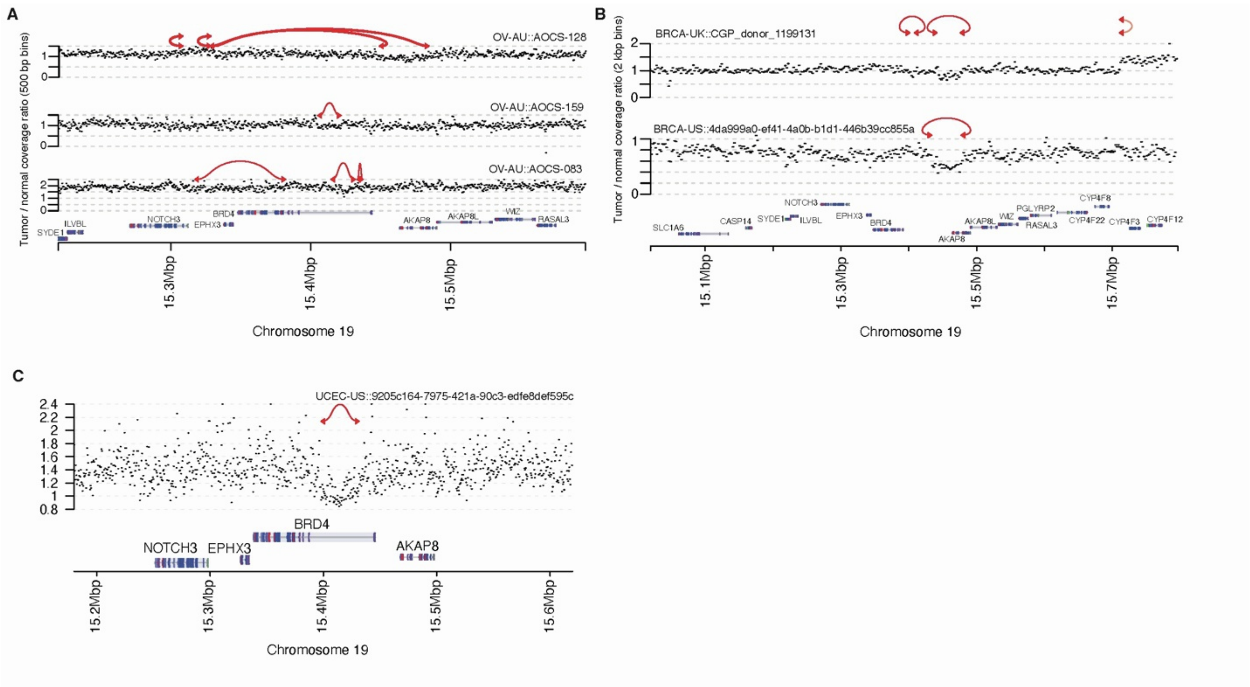

Figure S2. Tumors with *BRD4*-locus breakpoints but with no corresponding SCNAs, related to Figure 1. Tumor/normal coverage ratios from binned read-depth signals (y-axis) and rearrangements (red lines; arrows indicate breakpoint orientation) for tumors without a called SCNA from read-depth only signal for **(A)** ovarian cancer (n=3), **(B)** breast cancer (n=2), and **(C)** endometrial cancer (n=1).

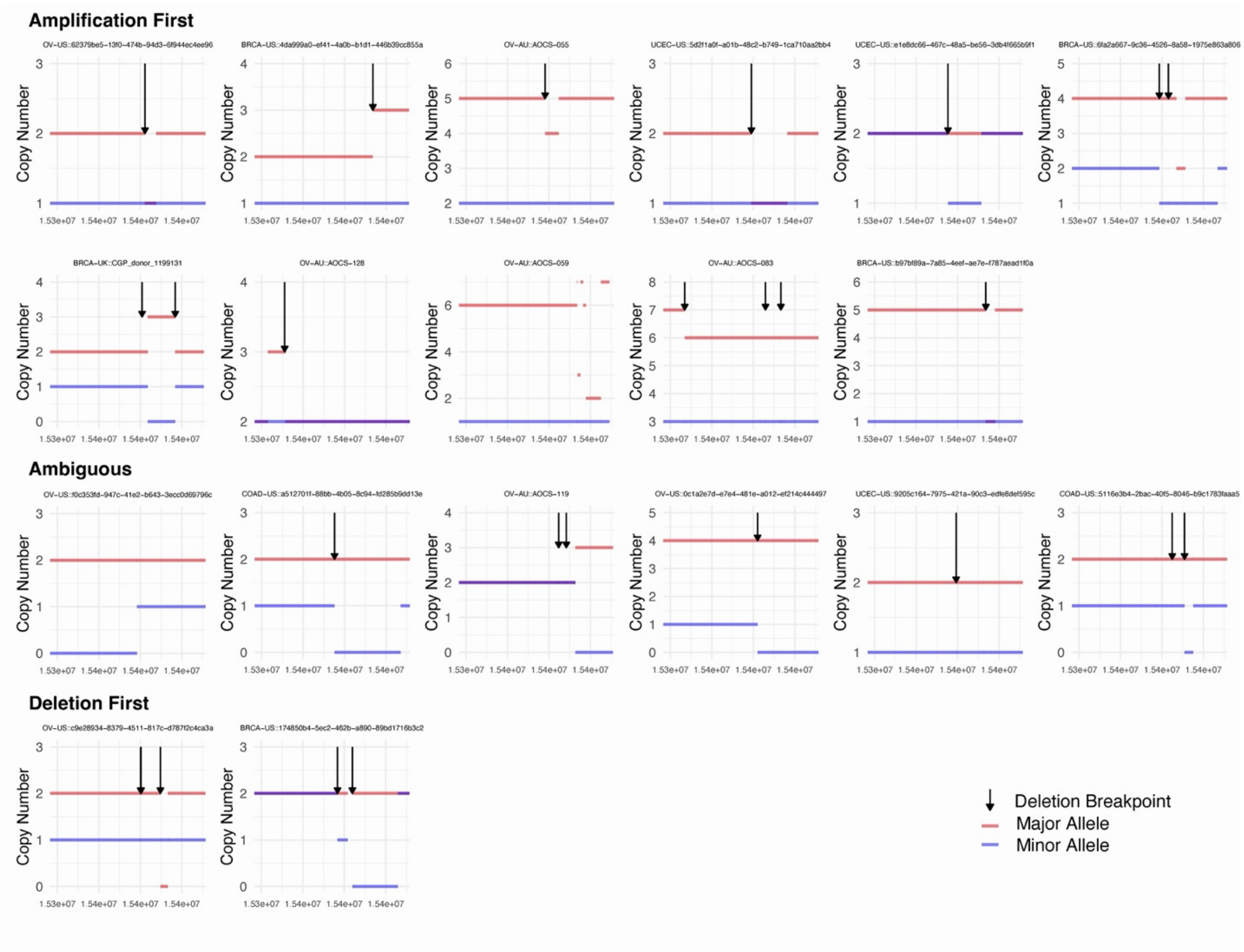

Figure S3. Allelic copy number analysis reveals most likely event timing, related to Figure 1. SCNA temporal ordering using phased allele-specific copy number for samples with both amplification and deletion. Red and blue distinguish the major and minor alleles, and black arrows denote SV breakpoints called by the ICGC-PCAWG consensus SV pipeline. In several instances, the deletion effect is too small to register a change in copy number but is confirmed to affect read depth (plotted in **Figure S2**). SCNA copy losses are occasionally unaccompanied by a consensus-called breakpoint.

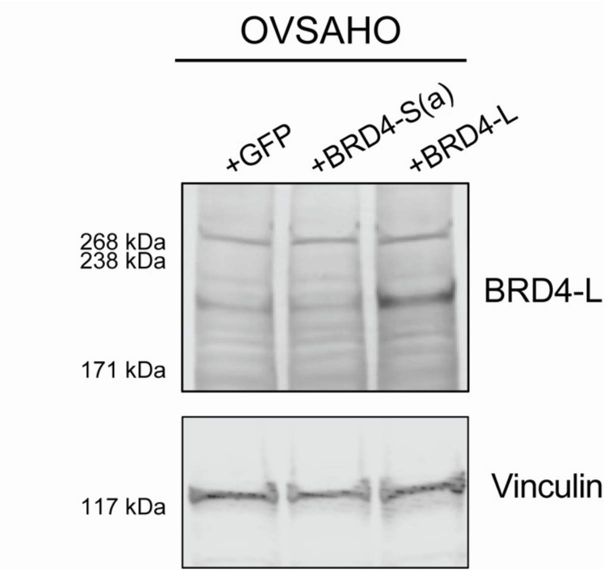

Figure S4. Isoform-specific detection of BRD4 protein, related to Figure 3. Detection of BRD4-L protein expression in total lysates from OVSAHO cells overexpressing GFP control, BRD4-S(a), or BRD4-L using an isoform-specific antibody.

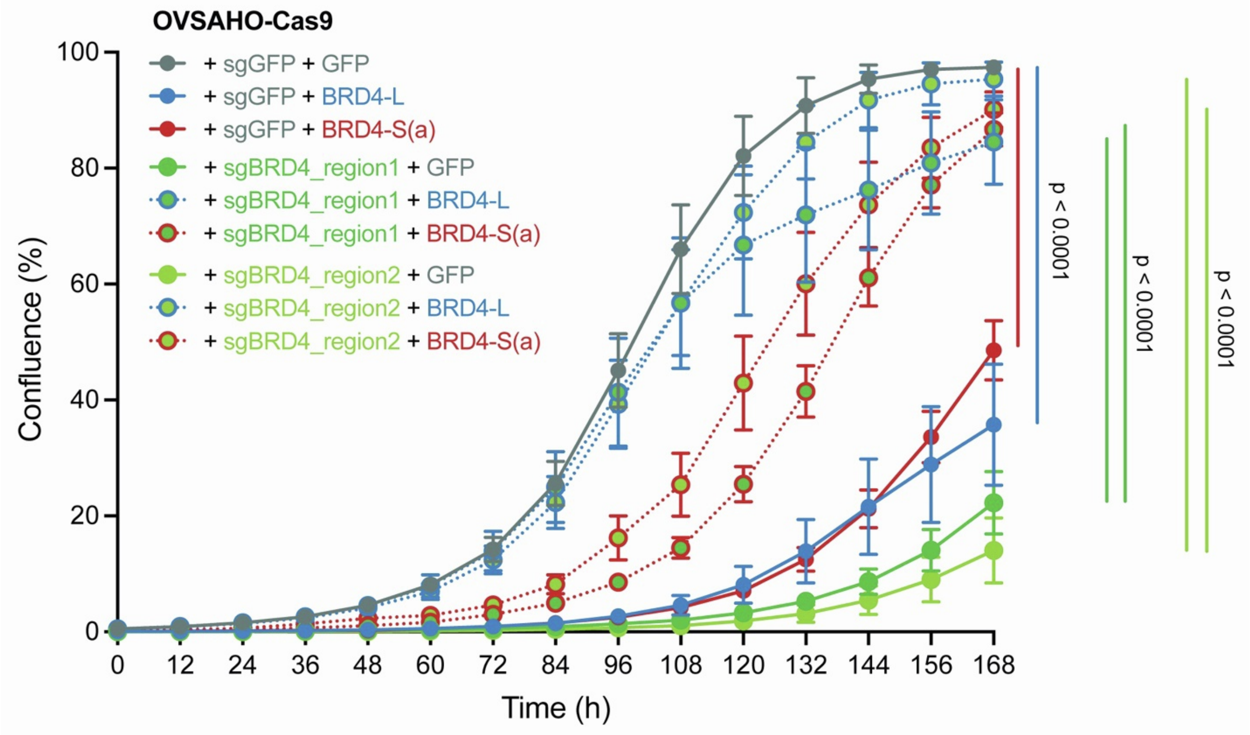

Figure S5. Quantification of cell confluency in OVSAHO-Cas9 cells over-expressing BRD4-L or BRD4-S(a) isoforms and rescue of proliferation with CRISPR-Cas9-mediated sgRNA cutting of *BRD4* regulatory regions, related to Figure 3. The effect of BRD4 over-expression and/or depletion on cell confluence is shown relative to sgGFP + GFP control. Mean and standard error of three replicates. Two-way ANOVA, followed by Dunnett post-tests, controlling the Family-wise alpha threshold and confidence level. P-values of the final time point are annotated.
